# Identifying sources, pathways and risk drivers in ecosystems of Japanese Encephalitis in an epidemic-prone north Indian district

**DOI:** 10.1371/journal.pone.0175745

**Published:** 2017-05-02

**Authors:** Manish Kakkar, Sanjay Chaturvedi, Vijay Kumar Saxena, Tapan N. Dhole, Ashok Kumar, Elizabeth T. Rogawski, Syed Abbas, Vidya V. Venkataramanan, Pranab Chatterjee

**Affiliations:** 1 Public Health Foundation of India, Gurgaon, Haryana, India; 2 Department of Community Medicine, University College of Medical Sciences, Delhi, India; 3 Public Health Foundation of India, Gurgaon, Haryana, India; 4 Department of Microbiology, Sanjay Gandhi Post Graduate Institute of Medical Sciences, Lucknow, Uttar Pradesh, India; 5 Department of Veterinary Public Health, Indian Veterinary Research Institute, Izatnagar, Uttar Pradesh, India; University of Texas Medical Branch at Galveston, UNITED STATES

## Abstract

Japanese Encephalitis (JE) has caused repeated outbreaks in endemic pockets of India. This study was conducted in Kushinagar, a highly endemic district, to understand the human-animal-ecosystem interactions, and the drivers that influence disease transmission. Utilizing the ecosystems approach, a cross-sectional, descriptive study, employing mixed methods design was employed. Four villages (two with pig-rearing and two without) were randomly selected from a high, a medium and a low burden (based on case counts) block of Kushinagar. Children, pigs and vectors were sampled from these villages. A qualitative arm was incorporated to explain the findings from the quantitative surveys. All human serum samples were screened for JE-specific IgM using MAC ELISA and negative samples for JE RNA by rRT-PCR in peripheral blood mononuclear cells. In pigs, IgG ELISA and rRT-PCR for viral RNA were used. Of the 242 children tested, 24 tested positive by either rRT-PCR or MAC ELISA; in pigs, 38 out of the 51 pigs were positive. Of the known vectors, *Culex vishnui* was most commonly isolated across all biotopes. Analysis of 15 blood meals revealed human blood in 10 samples. Univariable analysis showed that gender, religion, lack of indoor residual spraying of insecticides in the past year, indoor vector density (all species), and not being vaccinated against JE in children were significantly associated with JE positivity. In multivariate analysis, only male gender remained as a significant risk factor. Based on previous estimates of symptomatic: asymptomatic cases of JE, we estimate that there should have been 618 cases from Kushinagar, although only 139 were reported. Vaccination of children and vector control measures emerged as major control activities; they had very poor coverage in the studied villages. In addition, lack of awareness about the cause of JE, lack of faith in the conventional medical healthcare system and multiple referral levels causing delay in diagnosis and treatment emerged as factors likely to result in adverse clinical outcomes.

## Introduction

Japanese Encephalitis (JE) is a mosquito-borne flavivirus that causes neurological infection in humans. Without early diagnosis and management, it may have mortality rates of 15–30%, and up to half of the survivors may have permanent, residual neuropsychiatric sequelae [1–4]. Although the first case of JE in India was reported in 1955, [5] it was not until 1978–79, when routine monitoring was started, that the real magnitude was ascertained. Between 1978 and 2007, 103,389 cases of JE or Acute Encephalitis Syndrome (AES) have been reported from India, with 33,729 deaths (case fatality rate 32.6%). [6] From 2010 to 2014, Uttar Pradesh (UP) accounted for 42% of all AES cases and 22% of all JE cases reported from the country. [7] Endemic areas experience cyclical epidemics associated with high mortality. [8,9].

Despite growing concerns over the emergence of JE in India, it remains poorly understood, mainly because the problem has been approached in a compartmentalized manner, with human health, animal health, environment, socio-economic factors, policy design and implementation being examined in isolated silos. This has resulted into sector-specific interventions like vaccination and segregation of piggeries, which have not resulted in a significant reduction in the incidence of JE/AES.

There is a need to study the disease and its drivers in an integrated, transdisciplinary framework for a holistic understanding of the complex interplay of factors and to design effective interventions. [10] The present study was conducted in a high endemic district of Uttar Pradesh (UP) to understand the human-animal-ecosystem interactions, as well as the social and the environmental factors that influence disease transmission in this region.

## Materials and methods

**Fig 1:**
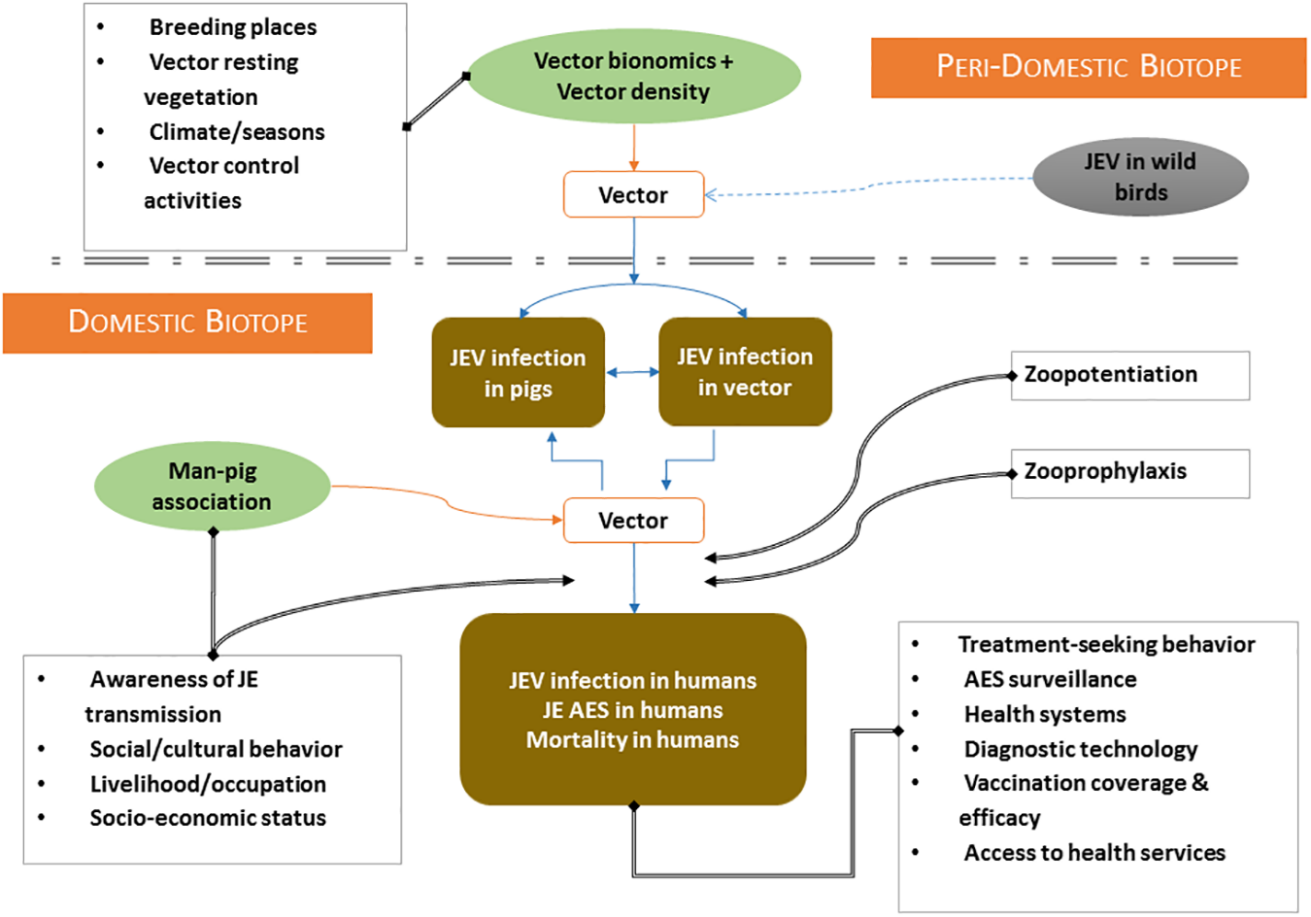
Conceptual framework of Japanese Encephalitis transmission developed by the EcoHealth Research Core Group.

A cross-sectional, descriptive study, employing a mixed methods design was conducted in the Kushinagar district of UP, between July 2012 and October 2014. A multidisciplinary team of researchers, the EcoHealth Research Core Group (ERCG), created a conceptual framework (Fig 1) illustrating typical elements of JE transmission, infection, and outcomes in the setting of an endemic North Indian village ecosystem based on a literature review, expert knowledge and the findings from a small exploratory study.

The ecosystem approach focused on the two subsystems of JE: the domestic biotope and the peri-domestic biotope. The domestic biotope was defined as the one in which humans reside, that is, within and around the immediate household settings, comprising of human houses and animal shelters, with characteristic cohabitation of humans and domesticated animals. The domestic biotope was further divided into indoor and immediate outdoor biotopes. The peri-domestic biotope, defined as the area around the domestic biotope, comprised of crop vegetation, including rice/paddy fields, and other land and water bodies (such as ponds). The peridomestic biotope sometimes extended for several miles, and was the main connecting agro-ecosystem between villages. There were sparse patches of wastelands which could serve as seasonal breeding places for mosquitoes.

### Human sampling

Published data from endemic villages in South India have suggested JE-specific IgM is demonstrable in ~10% children. [11] Considering Kushingar to be a highly endemic district, and assuming the anticipated prevalence of JE-specific IgM to be 15%, minimal size of a random sample, at 95% confidence level, with absolute precision of 5%, power of 80% and alpha of 5% was computed to be 196 children.

Based on retrospective analysis of AES cases reported, blocks in Kushinagar were stratified into high, medium and low burden tertiles of endemicity, and one block was selected randomly from each of these stratum; then, two villages with pig rearing and two without pig rearing were selected randomly from each of the blocks. According to the 2001 census of India, there were 1572 villages and 426,064 households in Kushinagar, with an average of 271 households per village [12]. Using systematic random sampling, 5% of the village households were enrolled for the survey and blood-draws. Whole blood and sera were collected from all healthy children aged 1 to 15 years in these households. All serum samples were screened for JE-specific IgM using MAC ELISA. [13] Samples negative for anti-JEV IgM were tested for the presence of JE RNA by real time reverse transcription polymerase chain reaction (rRT-PCR) in peripheral blood mononuclear cells (PBMCs).[14] To rule out post-vaccination IgM antibodies, children vaccinated against JE in the preceding three months were excluded.

Household surveys for human and pig blood collection were conducted during peak transmission season (October-November) and entomological surveys for vector collection in pre-peak (June-July) and peak density seasons (August-September).

### Pig sampling

The pig survey was delinked from the household survey, since pig-rearing was seen in only one particular community in the villages. Considering a total universe of 300 pigs based on the initial surveys, for a JE seroprevalence of 20.6% as indicated by previous surveillance data, minimal size of a random sample, at 95% confidence level, with absolute precision of 10%, power of 80% and alpha of 5%, was computed to be 52 pigs [15]. Pigs older than 3 months of age were sampled. All pig blood samples were collected from anterior jugular vein in two aliquots–one used for separation of serum and other in anticoagulant as whole blood. Recent infection in pigs in sample villages was detected by testing pig serum samples for JEV specific antibodies. IgG ELISA in younger cohorts (pigs aged 3–8 months), that had lived through only one transmission cycle, was used as a proxy test for recent infection instead of using IgM MAC ELISA as the commercial kits for the latter were unavailable, and attempts to standardise an in-house test during the study period were unsuccessful. Pig whole blood samples were also tested for viral RNA in peripheral blood mononuclear cells (PBMC) using rRT-PCR.

### Entomological survey

Entomological survey involved both larval sampling and adult mosquito collection. In each village, before the start of entomological survey, data collectors interacted with village leaders to ascertain presence and location of prominent landmarks. A map of the village was drawn, showing approximate demarcation of the village area into domestic indoor, domestic outdoor and peri-domestic biotopes with important landmarks and vegetation types in and around the village.

#### Larval sampling

Mosquito larvae were collected using a standard larval ladle [16] from ground water collections. First, water samples were collected from around all the brick kilns, large water bodies (e.g. pond) and fallow land within 2 km radius of the village; small water collections around each landmark were considered to be the same source. Special attention was given to water hyacinth vegetation, known to facilitate breeding of mosquito larvae. Second, to ensure randomness and representativeness, yet feasibility, water samples were collected from 5% (every 20^th^ water collection) of the water collection around paddy fields and other sources of ground water collection (e.g. puddles and ditches) along a 500m strip around the village.

#### Adult mosquito collection

Adult mosquitoes were collected from domestic indoor, domestic outdoor and peri-domestic biotopes of all villages.

#### Domestic indoor collection

Each village was demarcated into three concentric zones (centre, medium and peripheral zones). Five houses meeting the selection criteria were selected (two each from periphery of the village and middle zone and one from the centre). Standard total catch by space spray method [16] was used to collect indoor resting mosquitoes in morning hours (6-8am) and vector density estimated as number per room density (PRD).

#### Domestic outdoor collection

Outdoor resting mosquitoes were collected from vegetation using the Hop Cage Method. Among patches of domestic outdoor vegetation, mosquitoes were collected from three patches (one each from the periphery, middle and central areas of the village).

#### Peri-domestic biotope collection

Based on the local agricultural practice, six types of crop vegetations were identified for mosquito collection: paddy, sugarcane, wheat, fodder plant, millet, and mustard. At least four fields (one from each of the four directions) from each type of vegetation were sampled using the BPD Hop Cage Method as outlined by Das [17]. Vector density was estimated as number per hop cage (PHC). Outdoor adult resting mosquito samples from the domestic and peri-domestic biotopes were collected in early morning hours before 10:00 am.

Geo-spatial data on land use/land cover for ecological assessment was collected during the entomological surveys.

### Qualitative methods

A qualitative arm was integrated into the methods to identify risk drivers that might have been missed during quantitative estimation. The knowledge, perceptions and practices of various stakeholders on JE/AES transmission, prevention, control and treatment during acute illness were mapped through 17 in-depth interviews (IDIs), and four focus group discussions (FGDs). Community, district, state and national stakeholder consultations were conducted for respondent validation and refinement of the emerging model. Synergy or divergence between the perceptions of different stakeholders were mapped through thematic analysis of transcripts.

### Analysis plan

Quantitative data were analysed by descriptive tabulations, followed by univariable and multivariable analyses using logistic regression to identify the significant risk drivers for JE infection. It was postulated that the presence of the identified risk drivers were positively associated with JE infection in children in the sample villages. For qualitative data, triangulation was done both across methods as well as across respondent groups. The grounded theory approach was used to explain the derived phenomena.

### Ethical considerations

Ethical clearances were obtained from the Public Health Foundation of India’s Institutional Ethics Committee and the Institute Animal Ethics Committee of the Indian Veterinary Research Institute (Approval #10, dated 2 Dec 2011, IVRI IAEC proceedings). Due approval was also obtained from the Health Ministry’s Screening Committee (HMSC). Written informed consent was obtained from all participants.

## Results

### Sample characteristics

The study included 125 households from 12 villages in three blocks. There were 38 households from Padrauna, 40 from Kaptanganj and 47 from Khadda. The median size of the households was seven members (IQR 5–9); the median number of rooms were two (IQR 2–4). Almost half of the respondents (49.4%) were men; it was a predominantly young population with the mean age being 23 years and 42% being aged under 15 years; amongst those older than 15 years, 64% self-reported as literate.

### JE positivity in children

Blood samples were obtained from 242 of the 363 eligible children (65%); 24 tested positive (9.9%) either for JE IgM or for JE RNA. Out of the positive cases, only two were positive by ELISA IgM and the rest were positive on rRT-PCR. Padrauna accounted for the highest number of JE positive children (n = 12), followed by Kaptanganj (n = 7) and Khadda (n = 5). There was a significant relationship between increasing age of the child and JE virus (JEV) positivity (χ^2^ = 25.79, df = 14, p = 0.027).

### JE positivity in pigs

Of 105 pigs in the sampled households, 56 were eligible for inclusion, and 51 (91%) were tested. Of them, 38 (74.5%) were positive for recent JEV infection using either ELISA (n = 35, 68.6%) or PCR (n = 8, 15.6%). A chi-square test for independence did not reveal a relationship between age of the pig and JEV positivity. The proportion of pigs positive for JE in the three blocks was not statistically significantly different.

### Vector survey and JE positivity

In course of the study a total of 448 sites were sampled, over 6,000 mosquitoes were collected, of which 2% belonged to the species of interest (known vectors: *Culex vishnui*, and *Cx*. *tritaeniorhynchus*; suspected vectors: *Cx*. *gelidus*, *Cx*. *epidesmus*, and *Cx*. *whitmorei*). *Cx*. *vishnui* was the most prevalent species in all three blocks (62%), followed by *Cx*. *whitmorei* (29%). In the first round, the highest number of mosquitoes from the species of interest was retrieved from Kaptanganj (18/41), followed by Khadda (14/41) and Padrauna (9/41). In the second round, Khadda (47/60) accounted for the highest number of mosquitoes belonging to the species of interest, followed by Kaptanganj (9/60) and Padrauna (4/60). *Cx*. *vishnui* was the commonest isolate in both the rounds (34/41 in the first and 28/60 in the second). Further details of vector sampling results are provided in S1 Table, S2 Table and S3 Table in the supporting information.

JEV positivity was found in three pools of mosquito species that were not known or suspected vectors. Two of these pools were from Kaptanganj and consisted of 4 and 10 mosquitoes; the third positive pool was from Khadda and consisted of 53 mosquitoes. The number of villages with more than one vector species increased from the first round (three: Bahadurganj, Amdiha, Gajara) to the second one (five: Amdiha, Gajara, Belwa Jungal, Bulahwa, Chamardiha). Domestic indoor densities of vectors tended to be higher in villages with higher man:bovine ratios, although such correlations were not statistically significant. Vector density at the domestic outdoor sites was 1.5–2 times that in the peri-domestic sites. Overall, the vector density in the outdoor biotope for the three blocks combined was 0.24 mosquitoes/10 hop cages for the species of interest and 2.73 mosquitoes/10 hop cages for all species combined; the corresponding densities in the peri-domestic biotope were 0.13 and 0.78 mosquitoes/10 hop cages. Analysis of 15 blood meals from vectors in five villages in three blocks (in all three biotopes) revealed human blood in 10 samples (seven in domestic indoor, two in domestic outdoor and one in peridomestic biotopes respectively). Human blood meal was detected from *Cx*. *vishnui*, *Cx whitmorei* and *Cx gelidus*. Details of the vector density across the different biotopes, in different rounds of data collection are outlined in Table 1 below. More details of vector samples are provided in the S1, S2 and S3 Tables provided as supporting information.

**Table 1:** Vector density in the different biotopes.

District	Domestic Indoor Biotope						Domestic Outdoor and Peri-Domestic Biotope					
	Species of Interest density (No./room)			Other species density (No./room)			Species of Interest density (per 10 hop cages)			Other species density (per 10 hop cages)		
	R1	R2	R1+R2	R1	R2	R1+R2	R1	R2	R1+R2	R1	R2	R1+R2
**Padrauna**	0.54	0.10	0.27	77.23	56.45	64.64	0.05	0.04	0.04	0.18	1.27	0.77
**Kaptanganj**	0.79	0.30	0.50	30.71	19.40	24.06	0.30	0.07	0.15	1.35	0.90	1.06
**Khadda**	0.50	1.45	1.09	30.00	93.50	69.69	0.26	0.32	0.30	4.16	1.18	2.24

### Drivers of JE virus transmission

The outcome variable was defined as JEV infection status (positive or negative). Univariable analysis showed that gender, religion, lack of indoor residual spraying of insecticides in the past year, indoor vector density (all species), and not being vaccinated against JE in children were significantly associated with JE positivity (Table 2).

**Table 2:** Univariable analysis of drivers of JE virus infection in children (1–15 years).

Parameter	OR	95% CI
**A. DOMESTIC BIOTOPE**		
**1. Demographics**		
Gender (Male compared to female)	4.32	1.56–12.01
Religion (Muslim compared to Hindu)	2.30	1.03–5.14
**2. Livestock Ownership and Human-Animal Contact**		
Man: animal ratio (log; per 1 log increase)	2.03	0.40–10.21
Man:bovine ratio (log; per 1 log increase)	2.43	0.71–8.29
Livestock Ownership	0.82	0.43–1.56
Village pig ownership	1.02	0.38–2.74
Village pig positivity (per 10% increase in pig-owning villages)	0.85	0.61–1.19
Bovines sleeping <5m from where humans sleep versus those that sleep >5m	0.53	0.12–2.31
**3. Vector Control and Density**		
Mosquito spraying in the past 1 year	0.31	0.19–0.50
Indoor Vector Density		
R1 all mosquitoes		
High	5.47	2.94–10.18
Medium	4.73	2.06–10.86
Low	1	—
1 unit increase	5.03	2.52–10.04
R2 all mosquitoes		
High	0.49	0.1–2.55
Medium	1.59	0.53 –.84
Low	1	—
1 unit increase	0.76	0.40–1.45
Outdoor Vector Density		
R1 species of interest		
High	0.22	0.08–0.57
Medium	0.62	0.29–1.31
Low	1	—
1 unit increase	0.52	0.35–0.76
R2 species of interest		
High	0.5	0.16–1.55
Medium	0.19	0.09–0.41
Low	1	—
1 unit increase	0.51	0.19–1.35
*Cx*. *vishnui* outdoor R1		
High	0.22	0.08–0.57
Medium	0.62	0.29–1.31
Low	1	—
1 unit increase	0.52	0.35–0.76
*Cx*. *vishnui* outdoor R2		
High	0.42	0.16–1.07
Low	1	—
**4. Vaccination Status**		
Proportion vaccinated in villages (per 10% increase)	0.29	0.16–0.53
**B. PERI-DOMESTIC BIOTOPE**		
**1. Location (Block)**		
Padrauna	3.28	1.01–10.65
Kaptanganj	1.81	0.3–8.98
Khadda	1	—
**2. Land use/land cover**		
Paddy 500 m (log; per 1 log increase)	9.8	0.29–333.18
Paddy 3 km (log; per 1 log increase)	21.7	0.13–3594.05

In multivariable analysis, only gender remained as a significant risk factor (OR 4.83, p<0.003) (Table 3).

**Table 3:** Multivariable analysis of drivers of JE virus infection in children (1–15 years).

Parameter	OR	95% CI	p-value
Location (Block)			
Padrauna	1.79	0.96–3.32	0.065
Kaptanganj	0.84	0.29–2.43	0.75
Khadda	1	—	
Proportion vaccinated in village (per 10% increase)	0.36	0.11–1.1723	0.09
Gender (Male compared to female)	4.83	1.68–13.88	0.003
Religion (Muslim compared to Hindu)	1.12	0.39–3.23	0.84
Mosquito spraying in the past 1 year	1.3	0.49–3.4	0.6

### Drivers of JE outcome

Results of the qualitative analysis of interviews with various community stakeholders indicated a lack of awareness about the disease, its causes, transmission, and prevention and control measures (Table 4).

**Table 4:** Main themes studied in qualitative analysis.

Stakeholders in IDIs	Core themes showing synergy across respondents	Core themes showing divergence across respondents
• Pig Owners • Utilizers of care of acute illness (AES) • Non-Utilizers of care of acute illness (AES) • Representatives of Non-Government Organizations (NGOs) • Health care providers (Human) • Health care providers (Veterinary) • District Level Providers: Human Health-1 + Veterinary Health-1	• JE/AES (Dimaghi Bukhar) is a deadly disease, but not a major health problem. • JE/AES is associated with general unhygienic conditions. No link with pigs. • Pig owners felt that pigs did not play a significant role in transmission of JE. • Minimal role of Accredited Social Health Activist (ASHA) or Auxiliary Nurse Modwife (ANM) (government health workers) in the first contact care of Acute Illness: First contact care usually provided by Non-Formal Prescriber in most cases. • No social or cultural resistance to JE vaccination or mosquito control activities. • No gender-based discrimination in the care of acute illness. • Non-utilization of funds available with Village Pradhan (Local Self Government).	• Awareness about JE/AES. • Incidence of JE/AES. • Coverage of JE vaccination. • Care of acute illness in health care system. • Training of human and veterinarian health functionaries. • Participation of NGOs
**Stakeholders in FGDs**		
• Farmers • Community leaders • Students (11–15 years)		

These are likely to influence disease outcomes. The only exceptions identified were that there did not appear to be any gender-based discrimination in the care of acute illness, and there were no perceived sociocultural barriers to JE vaccination or vector control programs. Qualitative analysis of drivers of JE outcomes complements the quantitative findings, and is forthcoming in a separate publication [18].

### Expected caseloads

Previous studies from India have ascertained that for every case of symptomatic JE, there are 200 patients who suffer from asymptomatic or subclinical disease [11]. Using the 2011 census population of Kushinagar (3,560,830) and the number expected to be under 15 years of age (1,246,573), the projected number of asymptomatic cases at the current prevalence of 9.9% is 123,627. Assuming the 1:200 ratio to be valid in this setting, the expected number of symptomatic or clinical cases of JE in Kushinagar should have been 618.

## Discussion

The endemicity of JE in India is attributable to several factors, including large number of people living in the vicinity of irrigated lands, high vector densities in endemic areas, dependence on pig farming, and meteorological conditions. [9,19,20] Given the contexts in which JE is typically transmitted in endemic areas, it is important to note that eco-epidemiological factors interact with the less well-defined socio-cultural drivers in a complex continuum to influence the overall disease epidemiology [21–25]. From an intervention perspective it is important to understand not only the factors that operate at the different levels but also the relationships that exist between them, some of which may be amenable to modifications to reduce the morbidity and mortality due to JE [24]. In this study, we investigated the different factors that drive JE transmission in a highly endemic JE ecosystem in Kushinagar.

Evidence of recent JE infection was seen in almost 10% of human (24/242) and 75% (38/51) of pig samples, with positive vector pools seen in 25% of the villages (3/12). The presence of a large pool of recently infected amplifier hosts (pigs), and the high density of vectors indicate the potential for high intensity transmission. The difference in infection rates between Khadda and Padrauna (OR 3.28, 95% CI 1.01–10.65) indicates that block level differences likely drive infection rates. We used an ecosystems approach to identify and explain the drivers for the likely emergence of such relatively high transmission of JE virus in Kushinagar.

Evidence from Southeast Asia puts seropositivity for JE in asymptomatic children between 5–8% and that among pigs at 30%. [26–29] The presence of 10% infected children and 75% infected pigs in our study indicates a much higher intensity of JE virus transmission, especially amongst the amplifying hosts. We estimated 618 symptomatic cases of JE in humans would have occurred in Kushinagar district alone in 2012. However, in that particular transmission season, only 139 cases were reported across UP, and 745 cases reported across India. [7] Aside from underreporting, this may also highlight the lacunae of the diagnostic tests employed. The National Vector Borne Disease Control Program (NVBDCP) recommends using IgM capture ELISA [30] for diagnosis and surveillance of JE. This test has been demonstrated to have high specificity, but poorer sensitivity, varying between 17–57%. [31] Consequently, there is a possibility that JE contributes to a greater proportion of AES cases than is identified at present. We employed rRT-PCR, which has been shown to have superior sensitivity and specificity [14,32] compared to IgM capture ELISA and RT-PCR, and this may have led to a higher yield for diagnosis of JE infection in this study. This calls for a closer scrutiny of the policy endorsing IgM ELISA as the first line diagnostic method. The clinical and public health significance of the detection of JEV RNA in PBMCs of a large proportion of samples, indicating latent infection [33], also merits investigation.

In the domestic outdoor biotope, vector biting and resting behaviour emerged as a driver, along with possible zoopotentiation due to the presence of cattle and other animals. Univariable analysis revealed that a higher density of *Cx*. *vishnui* and *Cx*. *tritaeneorhynchus* in the domestic outdoor biotope was associated with a significantly lower odds of JE in children. This finding, when analysed in combination with the fact that JE virus in human blood meal was isolated from species which are not known to transmit JEV, and that most of the blood meals of human source were seen in mosquitoes isolated from the domestic indoor biotope, raises some concerning hypotheses. The first is the possibility of species other than the ones which were identified to be of interest have a role to play in the transmission of JEV. The ability of *Culex pipiens*, *Culex orientalis* and *Aedes albopictus* to function as competent vectors for JEV have been documented recently.[34–36] The second is the possibility that the JE vectors, traditionally believed to be exophilic, exophagic, and zoophagic, are changing their behaviour in favour of endophily and endophagy. Further, finding of lower odds among children, despite higher vector density in domestic outdoor biotope, may indicate lower exposure of children to mosquito bite, as they sleep indoors. Limited blood meal analysis of adult mosquitoes further supported the possibility of opportunistic endophilic and endophagic behaviour in the vectors. Although conventional wisdom dictates that the vectors associated with JE transmission are strongly attracted to cattle and are exophilic and exophagic in nature, there has been emerging evidence, especially from highly endemic areas, which indicate that *Culex* mosquitoes may exhibit endophilism. [37] With indications of opportunistic endophilism, some locations in the village may be targeted for selective use of Indoor Residual Spraying of pesticides (IRS), especially in and around households which have domestic animals (for example, cattle) that may contribute to zoopotentiation. [38].

Cattle is believed to provide zooprophylaxis by attracting vectors for blood meals, but effective zooprophylaxis occurs only when a favourable man:animal ratio exists. If this ratio is not achieved, then there is the possibility of a detrimental zoopotentiating effect instead. [39,40] We found a statistically significant negative correlation between the number of cows in a village and the density of mosquitoes belonging to the species of interest found indoors during the peak transmission season (*r*: -0.643, p = 0.024).

The role of pigs as amplifier hosts of the JE virus is well established. [20,41] The presence of JE infected children in both pig-owning and non-pig-owning villages indicates high transmission of the virus. Backyard pig-rearing, with poor protection from vector bites, in combination with poor awareness regarding the disease, its spread, prevention and control, resulted in the failure to recognise the role of pigs in the epidemiology of JE. Similar perceptions have been documented among pig-rearing communities of Bangladesh, who believe that diseases could be transmitted between pigs, but not from pigs to humans. [42] Vectors could play a critical role in maintaining high JE infection rates in non-pig-rearing villages. Culicine mosquitoes have been shown to have mean flight range of 4.4 km, with maximum flight ranges of up to 12 km being documented in some settings.[43] Studies have implicated meteorological conditions and vector populations as critical to the occurrence of JE infection in areas without significant pig populations. [22,44] In Australia, it has been seen that herons and egrets are potential sources from where the vectors may acquire the JEV;[45] such a scenario, though not entirely unlikely in the Indian setting, is probably not a major driver. The proximity of villages and the feral nature of pigs which have been documented to have wide wandering ranges, in combination with extensive vector flight ranges, is likely to drive the intense transmission of JEV infection in children even in the villages without any pig-rearing activities. This further reinforces the needs to limit access of vectors to both the amplifying hosts, as well as man.

Some experts have recommended moving pig shelters away from human habitats to reduce vector contact with them, but in the Indian context this would be practically impossible. [20,45] Proximity of villages and flight range of vectors are likely to offset any gains. There is an urgent need for a targeted intervention, such as covering of pig pens with Insecticide Treated Nets (ITNs). Previous studies have found that deployment of ITNs was associated with a sharp reduction in JE seroconversion in both pigs and human beings in endemic areas of India despite no significant reduction in outdoor vector densities. [38].

Another potential driver of JE infection, which functioned at the domestic biotope was the use of bed nets that were not impregnated with insecticides and IRS in the past year. The National Institute of Communicable Diseases (NICD) does not recommend IRS for control of JE vectors. [46] However, our study found that children from households that reported spraying in the past year were less likely to have JE infection. Furthermore, Padrauna and Kaptanganj blocks had the lowest incidence of IRS activities and also accounted for a higher number of JE-positive children. This calls for greater scrutiny of the potential benefits of IRS for JE vector control, especially given very low IRS coverage in all the sampled households (9%).

Although self-reported mosquito net usage was found to be high across all three blocks, none of these nets were insecticide impregnated, which may account for the failure to prevent JE infection. This is a major gap from a programmatic perspective as there is ample evidence that proper use of ITNs lowers the risk of acquiring JE infection. [38] The current study, however, did not observe bed net use patterns to ascertain whether they were being used properly; it has been shown that despite high usage rates, effective protection offered may be low if there is inconsistent or improper use of bed nets. [47,48] Future studies could be designed to address this lacuna of the current enquiry.

In the peridomestic biotope, vegetation type, mixed cropping, and vector behaviour emerged as potential drivers of JE infection. Gender was an important driver, possibly due to a higher likelihood of male children spending more time outdoors at dusk, thus being at a higher risk of being bitten by the vectors. [8].

A statistically non-significant but positive relationship between area under paddy cultivation and JE case load in the three blocks was observed. Although it has been difficult to establish the relationship between individual types of land cover and JE case loads, incidence of JE is known to be associated with extent of irrigated land. [49,50] It is likely that a situation analogous to the malaria “paddies paradox”, is occurring in this case: intensive agriculture leads to increased vector density, but in an area which is already experiencing intense and stable disease transmission, it is unlikely to cause a significant surge in the number of cases. [51] Emerging evidence suggests that mixed and multiple cropping system may have a protective effect. [52] In the case of the studied blocks, Khadda, which had the lowest JE case load, also had the widest array of agricultural products.

With respect to the systems drivers, vaccination emerged as a major protective factor in children. There was a statistically significant difference in the vaccination rates across the blocks (χ^2^ = 85.01, df = 2, p<0.001) with all vaccinated children being from Khadda. None of the vaccinated children had tested positive for JE infection, indicating that vaccination could be the mainstay of preventive activities. However, overall, only 13% (40/314) of the studied children reported being vaccinated against JE. This is in stark contrast to the reported figures, with coverage between 2006 to 2009 being reported to be 79%. [53] This is also much lower compared to the findings of the Coverage Evaluation Survey (CES) 2009, which estimated that 61% of the children were fully immunized under the universal immunization program. [54] This divergence was also noted in the qualitative arm, where health functionaries believed that there was high coverage for JE vaccination, whilst the community failed to reinforce the assertion.

This calls for reforms on two counts: first, in the methodology of ascertaining coverage, especially for intense, campaign-mode vaccination as in the case of JE; and second, in providing better integration of JE vaccination with routine and supplementary immunization (SIA) activities. Thus, the push for increasing vaccination against JE in campaign mode, especially in endemic districts is welcome, but, in the absence of a robust coverage evaluation system, and a surveillance system, it is likely to result in sub-optimal outcomes.

We did not observe statistically significant difference in JE infection in children belonging to different socioeconomic strata. While the flight range of mosquitoes, high vector densities, and the high animal ownership patterns (40–50% animal ownership across all blocks) could explain the absence of a difference of JE infection across different socioeconomic status groups, this relationship needs further investigation.

Studies have repeatedly shown the high risk of adverse clinical outcomes in JE. Mortality has been shown to be in the range of 15%-30%, with long term sequelae being noted in 20%-50% patients followed up post-discharge [1–4]. Early diagnosis has been shown to be a key element in preventing incidence of fatal outcomes or long term sequelae in survivors with JE infection [55]. The qualitative arm provided insights into the drivers that may influence disease outcomes. Lack of awareness about the JE/AES risk factors, causes, prevention, control, and treatment, stood out as drivers of possible adverse clinical outcomes in patients of JE. This indicates that enforcement of information education and communication (IEC) activities needs to be given priority before implementing behaviour change communication (BCC) campaigns.

Acute care was fraught with systemic failures. Peripheral health care workers were not accessible and lacked credibility. Consequently, patients had to go through a series of referral steps, often resulting in unnecessary investigations and medications. Patients usually approached a non-formal practitioner, then, on worsening, got referred to private practitioners, who sent them to primary health centres, and eventually the district hospital or the local medical college. The time lost increased the risks of unfavourable outcomes. Faith in service providers and perceived effectiveness of available services are amongst the major determinants of health care seeking decisions made by the studied participants. Strengthening public-private partnerships at local levels and building a culture of rational use of diagnostic tools and medicines may help in addressing these problems.

**Fig 2:**
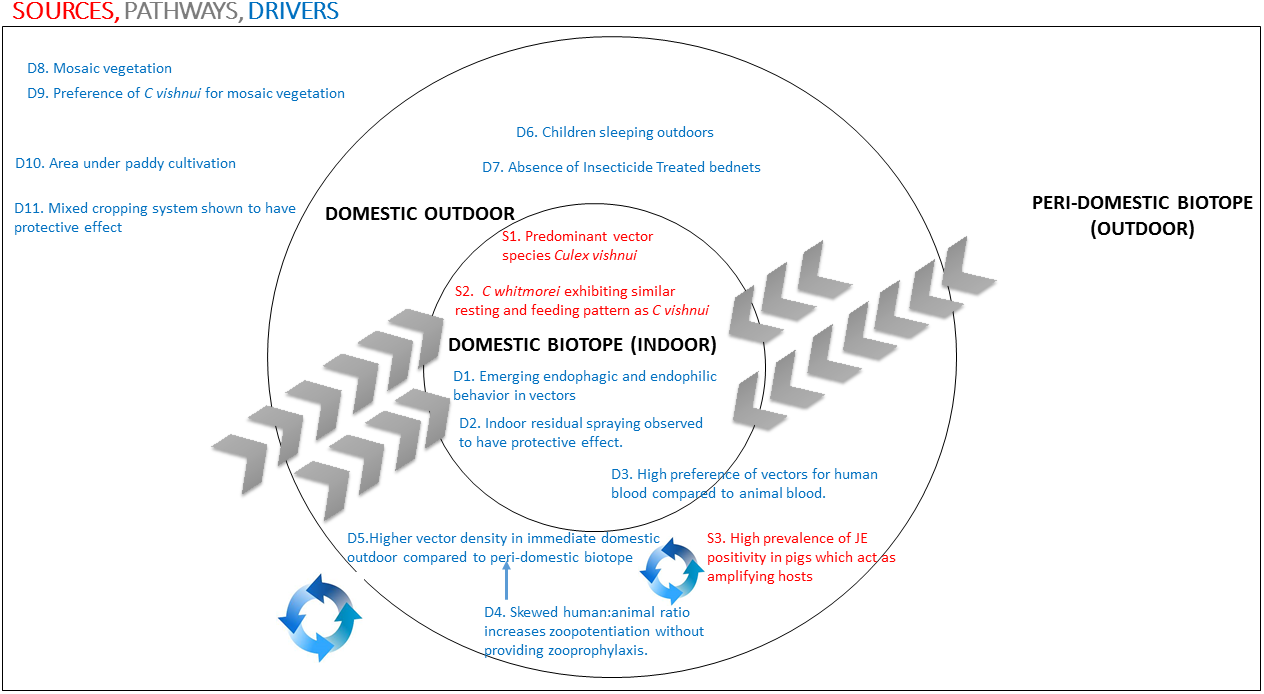
Sources, pathways and drivers of JE infection in ecosystems of high endemicity.

Based on the initial conceptual model (Fig 1 above) and the findings from the qualitative and quantitative arms of the study, a refined model was constructed, showing the sources, pathways and drivers of JE infection in ecosystems of high endemicity, as in the case of Kushinagar (Fig 2 below).

## Limitations

Limitations of the currently available serological tests (MAC ELISA) in detection of acute infection in symptomatic JE cases have been recently documented [56]; while we tried to address this by using rRT-PCR, this could explain the low positivity in our samples. [57] We did not investigate the role of wild birds and instead focused on avenues which have greater programmatic relevance from an intervention perspective.

Given its broad and holistic approach, EcoHealth research captures the complexities of the real world of disease prevention and control by covering more ground, if not depth. This may lead to loss of statistical power. In the real world situation, where often many of these hypotheses are overlapping and inter-related, it is rare that all but one hypothesis are rejected, leading to a summary understanding of the underlying factors. JE or other vector borne diseases, characterized by a complex ecology, thus need to be described through multiple hypotheses. In reality, it is likely, as we have shown in this study, that the outcomes are driven by a “composite of forces”; [58] multiple hypotheses, representing a portion of this composite, need to be evaluated so as to find the best fit. From programmatic angles, the onus is on finding those drivers which are most amenable to intervention. These considerations, which are the unique strengths of an EcoHealth approach, cannot always be accommodated within the boundaries of significance imposed by conventional statistics.

Despite the lack of statistically significant relationships, several associations have emerged which open up avenues of further enquiry, including several potential points of intervention to deal with the burden of JE. Further studies could concentrate on adopting study designs which help in establishing the strength of these associations, and if possible, identify causal relationships, if any.

## Conclusions

The first biotope-based study of the sources, pathways, and drivers of JE in a highly endemic district of India revealed the predominant drivers of JE infection in children as well as amplifier hosts (pigs). High vector density, emerging evidence of endophagic/endophilic behaviour in mosquito vectors, role of IRS in preventing infection, high prevalence of JEV infection in amplifier hosts (pigs), poor coverage of JE vaccination in sampled children, and an overall lack of awareness of the disease were identified as major drivers of infection and adverse clinical outcomes. Based on the JE infection rate, we estimated that in 2012, there should have been 618 cases of JE in Kushinagar. However, in 2012, there were 745 cases of JE reported from India. This mismatch could be explained by underreporting, as well as the choice of diagnostic tests, which have high specificity but poor sensitivity. These results provide valuable insights for program managers to design transdisciplinary interventions to combat JE infections in the community. Further, this study provides a template to study JE and other vector borne diseases through the agent-host-vector-environment interactions in the context of different biotopes.

## Supporting information

S1 Table Number of mosquitoes collected from the domestic indoor biotope in the two rounds. (DOCX)

S2 Table Number of mosquitoes collected from the domestic outdoor biotope. (DOCX)

S3 Table Number of mosquitoes collected from the peridomestic biotope. (DOCX)

S4 Table List of quantitative and qualitative data collection methods, sampling scheme and study population. (DOCX)

S5 Table Main variables studied in quantitative analysis. (DOCX)

S6 Table List of drivers by biotopes. (DOCX)

S7 Table List of villages by pig ownership. (DOCX)

S8 Table Man:Animal ratios in study villages. (DOCX)

S1 File Details of ethical approvals. (DOCX)

## References

[1] Ghosh D, Basu A. Japanese encephalitis-a pathological and clinical perspective. PLoS Negl Trop Dis. Public Library of Science; 2009;3: e437 doi: 10.1371/journal.pntd.0000437 19787040 10.1371/journal.pntd.0000437 PMC2745699

[2] Pieper SJ, Kurland LT. Sequelae of Japanese B and mumps encephalitis: recent follow-up of patients affected in 1947–1948 epidemic on Guam. Am J Trop Med Hyg. 1958;7: 481–90. Available: http://www.ncbi.nlm.nih.gov/pubmed/13571562 13571562

[3] Kakoti G, Dutta P, Ram Das B, Borah J, Mahanta J. Clinical profile and outcome of Japanese encephalitis in children admitted with acute encephalitis syndrome. Biomed Res Int. Hindawi Publishing Corporation; 2013;2013: 152656 doi: 10.1155/2013/152656 24490147 10.1155/2013/152656 PMC3891618

[4] Baruah HC, Biswas D, Patgiri D, Mahanta J. Clinical outcome and neurological sequelae in serologically confirmed cases of Japanese encephalitis patients in Assam, India. Indian Pediatr. 2002;39: 1143–8. Available: http://www.ncbi.nlm.nih.gov/pubmed/12522277 12522277

[5] Carey DE, Myers RM, Pavri KM. Japanese encephalitis studies in Vellore, South India. II. Antibody response of patients. Indian J Med Res. 1968;56: 1319–29. Available: http://www.ncbi.nlm.nih.gov/pubmed/4302907 4302907

[6] Dhillon GPS, Raina VK. Epidemiology of Japanese encephalitis in context with Indian scenario. J Indian Med Assoc. 2008;106: 660–3. Available: http://www.ncbi.nlm.nih.gov/pubmed/19552100 19552100

[7] National Vector Borne Disease Control Program. National Vector Borne Disease Control Program: Japanese Encephalitis [Internet]. 2015 [cited 24 Jul 2015]. Available: http://nvbdcp.gov.in/je-new.html

[8] Saxena V, Dhole TN. Preventive strategies for frequent outbreaks of Japanese encephalitis in Northern India. J Biosci. 2008;33: 505–14. Available: http://www.ncbi.nlm.nih.gov/pubmed/19208976 19208976 10.1007/s12038-008-0069-9

[9] Kumari R, Joshi PL. A review of Japanese encephalitis in Uttar Pradesh, India. WHO South-East Asia J Public Heal. 2012;1: 374–395. 10.4103/2224-3151.207040 28615603

[10] Singh A, Saxena SK, Srivastava AK, Mathur A. Japanese encephalitis: A persistent threat. Proceedings of the National Academy of Sciences India Section B—Biological Sciences. 2012.

[11] Gajanana A, Thenmozhi V, Samuel PP, Reuben R. A community-based study of subclinical flavivirus infections in children in an area of Tamil Nadu, India, where Japanese encephalitis is endemic. Bull World Health Organ. 1995;73: 237–44. Available: http://www.pubmedcentral.nih.gov/articlerender.fcgi?artid=2486759&tool=pmcentrez&rendertype=abstract 7743596 PMC2486759

[12] Census of India Website: Office of the Registrar General & Census Commissioner, India [Internet]. [cited 22 Jul 2015]. Available: http://censusindia.gov.in/

[13] Panbio Diagnostics. Japanese encephalitis-Dengue IgM combo ELISA test. In: Panbio Diagnostics [Internet]. 2008 [cited 4 Nov 2015] p. 6 Available: http://alere.co.jp/products02/panbio/japanese_encephalitis.pdf

[14] Saxena V, Mishra VK, Dhole TN. Evaluation of reverse-transcriptase PCR as a diagnostic tool to confirm Japanese encephalitis virus infection. Trans R Soc Trop Med Hyg. 2009;103: 403–6. doi: 10.1016/j.trstmh.2009.01.021 19249068 10.1016/j.trstmh.2009.01.021

[15] Geevarghese G, Shaikh BH, Jacog PG, Bhat HR. Monitoring Japanese encephalitis virus activity using domestic sentinel pigs in Mandya district, Karnataka state (India). Indian J Med Res. 1991;93: 140–2. Available: http://www.ncbi.nlm.nih.gov/pubmed/1657767 1657767

[16] WHO Division of Malaria and Other Parasitic Diseases. Manual on Practical Entomology in Malaria Part 1—Vector Bionomics and Organization of Anti-Malaria Activities [Internet]. 1st ed. Geneva: World Health Organization; 1975 Available: http://apps.who.int/iris/bitstream/10665/42481/1/WHO_OFFSET_13_(part1).pdf

[17] Das BP. BPD Hop cage method for effective JE vector surveillance In: Das Bina Pani, editor. Mosquito Vectors of Japanese Encephalitis Virus from Northern India. 1st ed. New Delhi: Springer India; 2013 pp. 43–60.

[18] Chaturvedi S, Sharma N, Kakkar M, Abbas S. Limited Understanding of Perceptions, Practices and Health seeking Behaviour constrain JE/AES Interventions in High Endemic District of North India: a qualitative enquiry. Connections for Health, Ecosystems and Society. Montreal: International Association for Ecology and Health; 2014 p. 207 Available: http://www.copeh-canada.org/upload/files/Abstract_Book_EcoHealth2014.pdf

[19] Kanojia PC, Shetty PS, Geevarghese G. A long-term study on vector abundance & seasonal prevalence in relation to the occurrence of Japanese encephalitis in Gorakhpur district, Uttar Pradesh. Indian J Med Res. 2003;117: 104–10. Available: http://www.ncbi.nlm.nih.gov/pubmed/14575175 14575175

[20] Solomon T. Control of Japanese encephalitis—within our grasp? N Engl J Med. 2006;355: 869–71. doi: 10.1056/NEJMp058263 16943399 10.1056/NEJMp058263

[21] Dhiman RC. Emerging Vector-Borne Zoonoses: Eco-Epidemiology and Public Health Implications in India. Front Public Heal. Frontiers; 2014;2: 168. 10.3389/fpubh.2014.00168 PMC4179687 25325052

[22] Tian H-Y, Bi P, Cazelles B, Zhou S, Huang S-Q, Yang J, et al How environmental conditions impact mosquito ecology and Japanese encephalitis: an eco-epidemiological approach. Environ Int. 2015;79: 17–24. doi: 10.1016/j.envint.2015.03.002 25771078 10.1016/j.envint.2015.03.002

[23] Ahmad A, Khan MU, Gogoi LJ, Kalita M, Sikdar AP, Pandey S, et al Japanese Encephalitis in Assam, India: Need to Increase Healthcare Workers’ Understanding to Improve Health Care. Gupta V, editor. PLoS One. Public Library of Science; 2015;10: e0135767 doi: 10.1371/journal.pone.0135767 26296212 10.1371/journal.pone.0135767 PMC4546657

[24] Burniston S, Okello AL, Khamlome B, Inthavong P, Gilbert J, Blacksell SD, et al Cultural drivers and health-seeking behaviours that impact on the transmission of pig-associated zoonoses in Lao People’s Democratic Republic. Infect Dis poverty. BioMed Central; 2015;4: 11 doi: 10.1186/2049-9957-4-11 25973203 10.1186/2049-9957-4-11 PMC4430026

[25] Holt HR, Inthavong P, Khamlome B, Blaszak K, Keokamphe C, Somoulay V, et al Endemicity of Zoonotic Diseases in Pigs and Humans in Lowland and Upland Lao PDR: Identification of Socio-cultural Risk Factors. Yang G-J, editor. PLoS Negl Trop Dis. Public Library of Science; 2016;10: e0003913 doi: 10.1371/journal.pntd.0003913 27070428 10.1371/journal.pntd.0003913 PMC4829221

[26] Yamanaka A, Mulyatno KC, Susilowati H, Hendrianto E, Utsumi T, Amin M, et al Prevalence of antibodies to Japanese encephalitis virus among pigs in Bali and East Java, Indonesia, 2008. Jpn J Infect Dis. 2010;63: 58–60. Available: http://www.ncbi.nlm.nih.gov/pubmed/20093765 20093765

[27] Ghimire S, Dhakal S, Ghimire NP, Joshi DD. Pig Sero-Survey and Farm Level Risk Factor Assessment for Japanese Encephalitis in Nepal. Int J Appl Sci Biotechnol. 2014;2: 311–314.

[28] Johnsen DO, Edelman R, Grossman RA, Muangman D, Pomsdhit J, Goould DJ. Study of Japanese Encephalitis virus in Chiangmai Valley, Thailand. V. Animal infections. Am J Epidemiol. 1974;100: 57–68. Available: http://aje.oxfordjournals.org/content/100/1/57 4367032 10.1093/oxfordjournals.aje.a112009

[29] Sugiyama T. Sero-epidemiological Studies on the Japanese Encephalitis. J Japanese Assoc Infect Dis. 2011;32: 255–269.

[30] Directorate of National Vector Borne Diseases Control Programme. Guidelines for surveillance of acute encephalitis syndrome (with special reference to Japanese Encephalitis) [Internet]. New Delhi; 2006. Available: http://nvbdcp.gov.in/Doc/AESguidelines.pdf

[31] Robinson JS, Featherstone D, Vasanthapuram R, Biggerstaff BJ, Desai A, Ramamurty N, et al Evaluation of three commercially available Japanese encephalitis virus IgM enzyme-linked immunosorbent assays. Am J Trop Med Hyg. 2010;83: 1146–55. doi: 10.4269/ajtmh.2010.10-0212 21036854 10.4269/ajtmh.2010.10-0212 PMC2963986

[32] Swami R, Ratho RK, Mishra B, Singh MP. Usefulness of RT-PCR for the diagnosis of Japanese encephalitis in clinical samples. Scand J Infect Dis. 2008;40: 815–20. doi: 10.1080/00365540802227102 18618334 10.1080/00365540802227102

[33] Sharma S, Mathur A, Prakash V, Kulshreshtha R, Kumar R, Chaturvedi UC. Japanese encephalitis virus latency in peripheral blood lymphocytes and recurrence of infection in children. Clin Exp Immunol. 2008;85: 85–89. 10.1111/j.1365-2249.1991.tb05687.x PMC1535705 1649022

[34] de Wispelaere M, Desprès P, Choumet V, Campbell G, Hills S, Fischer M, et al European Aedes albopictus and Culex pipiens are Competent Vectors for Japanese Encephalitis Virus. Turell MJ, editor. PLoS Negl Trop Dis. Public Library of Science; 2017;11: e0005294 doi: 10.1371/journal.pntd.0005294 28085881 10.1371/journal.pntd.0005294 PMC5268654

[35] Ravanini P, Huhtamo E, Ilaria V, Crobu MG, Nicosia A, Servino L, et al Japanese encephalitis virus RNA detected in Culex pipiens mosquitoes in Italy. Eurosurveillance. European Centre for Disease Prevention and Control (ECDC); 2012;17: 1–4. Available: http://www.eurosurveillance.org/ViewArticle.aspx?ArticleId=20221 10.2807/ese.17.28.20221-en 22835438

[36] Kim H, Cha G-W, Jeong YE, Lee W-G, Chang KS, Roh JY, et al Detection of Japanese Encephalitis Virus Genotype V in Culex orientalis and Culex pipiens (Diptera: Culicidae) in Korea. Baylis M, editor. PLoS One. Public Library of Science; 2015;10: e0116547 doi: 10.1371/journal.pone.0116547 25658839 10.1371/journal.pone.0116547 PMC4319795

[37] Kanojia PC, Geevarghese G. First report on high-degree endophilism in Culex tritaeniorhynchus (Diptera: Culicidae) in an area endemic for Japanese encephalitis. J Med Entomol. 2004;41: 994–6. Available: http://www.ncbi.nlm.nih.gov/pubmed/15535634 15535634 10.1603/0022-2585-41.5.994

[38] Dutta P, Khan SA, Khan AM, Borah J, Sarmah CK, Mahanta J. The effect of insecticide-treated mosquito nets (ITMNs) on Japanese encephalitis virus seroconversion in pigs and humans. Am J Trop Med Hyg. 2011;84: 466–72. doi: 10.4269/ajtmh.2011.10-0270 21363988 10.4269/ajtmh.2011.10-0270 PMC3042826

[39] Saul A. Zooprophylaxis or zoopotentiation: the outcome of introducing animals on vector transmission is highly dependent on the mosquito mortality while searching. Malar J. 2003;2: 32 doi: 10.1186/1475-2875-2-32 14565850 10.1186/1475-2875-2-32 PMC222927

[40] Mwandawiro C, Boots M, Tuno N, Suwonkerd W, Tsuda Y, Takagi M. Heterogeneity in the host preference of Japanese encephalitis vectors in Chiang Mai, northern Thailand. Trans R Soc Trop Med Hyg. 94: 238–42. Available: http://www.ncbi.nlm.nih.gov/pubmed/10974986 10974986 10.1016/s0035-9203(00)90303-1

[41] Erlanger TE, Weiss S, Keiser J, Utzinger J, Wiedenmayer K. Past, present, and future of Japanese encephalitis. Emerging Infectious Diseases. 2009. 10.3201/eid1501.080311 PMC2660690 19116041

[42] Khan SU, Salje H, Hannan A, Islam MA, Bhuyan AAM, Islam MA, et al Dynamics of Japanese encephalitis virus transmission among pigs in Northwest Bangladesh and the potential impact of pig vaccination. PLoS Negl Trop Dis. 2014;8: e3166 doi: 10.1371/journal.pntd.0003166 25255286 10.1371/journal.pntd.0003166 PMC4177832

[43] Bryan JH, O’Donnell MS, Berry G, Carvan T. Dispersal of adult female Culex annulirostris in Griffith, New South Wales, Australia: a further study. J Am Mosq Control Assoc. 1992;8: 398–403. Available: http://www.ncbi.nlm.nih.gov/pubmed/1474387 1474387

[44] Bi P, Zhang Y, Parton KA. Weather variables and Japanese encephalitis in the metropolitan area of Jinan city, China. J Infect. 2007;55: 551–556. doi: 10.1016/j.jinf.2007.07.004 17714787 10.1016/j.jinf.2007.07.004

[45] van-den-Hurk AF, Ritchie SA, Johansen CA, Mackenzie JS, Smith GA. Domestic pigs and Japanese encephalitis virus infection, Australia. Emerg Infect Dis. 2008; 10.3201/eid1411.071368 PMC2630726 18976557

[46] World Health Organization, Zoonosis Division National Institute of Communicable Disease. Guidelines for prevention and control of Japanese Encephalitis. [Internet]. New Delhi; 2006. Available: http://nicd.nic.in/writereaddata/linkimages/je3038660088.pdf

[47] Ngonghala CN, Del Valle SY, Zhao R, Mohammed-Awel J. Quantifying the impact of decay in bed-net efficacy on malaria transmission. J Theor Biol. 2014;363: 247–61. doi: 10.1016/j.jtbi.2014.08.018 25158163 10.1016/j.jtbi.2014.08.018 PMC4374367

[48] Stewart T, Marchand RP. Factors that affect the success and failure of InsecticideTreated Net Programs for malaria control in SE Asia and the Western Pacific [Internet]. 2001. Available: http://www.who.int/malaria/publications/atoz/itn_r62.pdf

[49] Robertson C, Pant DK, Joshi DD, Sharma M, Dahal M, Stephen C. Comparative spatial dynamics of Japanese encephalitis and acute encephalitis syndrome in Nepal. PLoS One. 2013;8: e66168 doi: 10.1371/journal.pone.0066168 23894277 10.1371/journal.pone.0066168 PMC3718805

[50] Impoinvil DE, Solomon T, Schluter WW, Rayamajhi A, Bichha RP, Shakya G, et al The spatial heterogeneity between Japanese encephalitis incidence distribution and environmental variables in Nepal. PLoS One. 2011;6: e22192 doi: 10.1371/journal.pone.0022192 21811573 10.1371/journal.pone.0022192 PMC3141013

[51] Ijumba JN, Lindsay SW. Impact of irrigation on malaria in Africa: paddies paradox. Med Vet Entomol. 2001;15: 1–11. Available: http://www.ncbi.nlm.nih.gov/pubmed/11297093 11297093 10.1046/j.1365-2915.2001.00279.x

[52] Keiser J, Maltese MF, Erlanger TE, Bos R, Tanner M, Singer BH, et al Effect of irrigated rice agriculture on Japanese encephalitis, including challenges and opportunities for integrated vector management. Acta Trop. 2005;95: 40–57. doi: 10.1016/j.actatropica.2005.04.012 15878762 10.1016/j.actatropica.2005.04.012

[53] Ministry of Health and Family Welfare, Government of India. Operational Guide Japanese Encephalitis Vaccination in India [Internet]. New Delhi; 2010. Available: http://www.iapcoi.com/hp/Dec25th/Guidelines-Japanese-Encephalitis,MoHFW,September2010%5B2%5D.pdf

[54] Ministry of Health and Family Welfare (India), ORG Centre for Social Research, United Nations Children’s Fund. India Coverage Evaluation Survey 2009–2010 [Internet]. 2010 [cited 10 Aug 2015]. Available: https://nrhm-mis.nic.in/SitePages/Pub_CoverageEvaluation.aspx#

[55] Luo D, Song J, Ying H, Yao R, Wang Z. Prognostic factors of early sequelae and fatal outcome of Japanese encephalitis. Southeast Asian J Trop Med Public Health. 1995;26: 694–8. Available: http://www.ncbi.nlm.nih.gov/pubmed/9139378 9139378

[56] Tiroumourougane S V, Raghava P, Srinivasan S. Japanese viral encephalitis. Postgr Med J. 2002;78: 205–215. 10.1136/pmj.78.918.205 PMC1742334 11930023

[57] Kakkar M, Dhole TN, Rogawski ET, Chaturvedi S. Public Health Laboratory Surveillance and Diagnosis of Japanese Encephalitis: Time to Revisit. Indian Pediatr. 2016;53: 33–5. Available: http://www.ncbi.nlm.nih.gov/pubmed/26840668 26840668 10.1007/s13312-016-0785-4

[58] Thompson Hobbs N, Hilborn R. Alternatives to statistical hypothesis testing in ecology: a guide to self-teaching. Ecol Appl. 2006;16: 5–19. 16705957 10.1890/04-0645

