# Supplementary material for: Identifying sources, pathways and risk drivers in ecosystems of Japanese Encephalitis in an epidemic-prone north Indian district
Source: PLoS One. 2017 May 2;12(5):e0175745. doi: 10.1371/journal.pone.0175745 (PMC5412994; doi:10.1371/journal.pone.0175745)
Supplement: S1 Table — (DOCX) [file pone.0175745.s001.docx]

## Table S1: Number of mosquitoes collected from the Domestic Indoor Biotope in the two rounds

|  |  | ***Culex vishnui*** | | ***Culex tritaeniorhynchus*** | | ***Culex gelidus*** | | ***Culex epidesmus*** | | ***Culex whitmorei*** | | **Other mosquito species** | | **Number of sites sampled** | |
| --- | --- | --- | --- | --- | --- | --- | --- | --- | --- | --- | --- | --- | --- | --- | --- |
|  |  | R1 | R2 | R1 | R2 | R1 | R2 | R1 | R2 | R1 | R2 | R1 | R2 | R1 | R2 |
| **Padrauna** | Bahadurganj | 0 | 0 | 1 | 0 | 1 | 0 | 0 | 1 | 0 | 0 | 235 | 181 | 3 | 5 |
|  | Sarrhie | 1 | 0 | 0 | 0 | 0 | 0 | 0 | 0 | 0 | 0 | 364 | 271 | 3 | 5 |
|  | Pipra Majra | 4 | 0 | 0 | 0 | 0 | 0 | 0 | 0 | 0 | 0 | 278 | 268 | 4 | 5 |
|  | Sidhua | 0 | 1 | 0 | 0 | 0 | 0 | 0 | 0 | 0 | 0 | 127 | 409 | 3 | 5 |
| **Kaptanganj** | Amdiha | 3 | 0 | 1 | 0 | 0 | 1 | 0 | 0 | 0 | 1 | 90 | 102 | 3 | 5 |
|  | Gajara | 1 | 1 | 0 | 0 | 0 | 1 | 0 | 0 | 2 | 0 | 0 | 124 | 3 | 5 |
|  | Ghurahupur | 2 | 1 | 0 | 0 | 0 | 0 | 0 | 0 | 0 | 1 | 170 | 20 | 3 | 5 |
|  | Magdiha | 2 | 0 | 0 | 0 | 0 | 0 | 0 | 0 | 0 | 0 | 170 | 142 | 5 | 5 |
| **Khadda** | Belwa Jungal | 3 | 3 | 0 | 0 | 0 | 0 | 0 | 0 | 0 | 1 | 90 | 752 | 3 | 5 |
|  | Bulahwa | 1 | 2 | 0 | 0 | 0 | 0 | 0 | 0 | 0 | 7 | 175 | 178 | 3 | 5 |
|  | Chamar Diha | 1 | 1 | 0 | 0 | 0 | 1 | 0 | 0 | 0 | 11 | 72 | 324 | 3 | 5 |
|  | Patkhauli | 1 | 3 | 0 | 0 | 0 | 0 | 0 | 0 | 0 | 0 | 23 | 616 | 3 | 5 |
|  |  | 19 | 12 | 2 | 0 | 1 | 3 | 0 | 1 | 2 | 21 | 1794 | 3387 | 39 | 60 |
