# Supplementary material for: Identifying sources, pathways and risk drivers in ecosystems of Japanese Encephalitis in an epidemic-prone north Indian district
Source: PLoS One. 2017 May 2;12(5):e0175745. doi: 10.1371/journal.pone.0175745 (PMC5412994; doi:10.1371/journal.pone.0175745)
Supplement: S2 Table — (DOCX) [file pone.0175745.s002.docx]

## Table S2: Number of mosquitoes collected from the domestic outdoor biotope

|  |  | ***Culex vishnui*** | | ***Culex tritaeniorhynchus*** | | ***Culex gelidus*** | | ***Culex epidesmus*** | | ***Culex whitmorei*** | | **Other mosquito species** | | **Number of sites sampled** | |
| --- | --- | --- | --- | --- | --- | --- | --- | --- | --- | --- | --- | --- | --- | --- | --- |
|  |  | R1 | R2 | R1 | R2 | R1 | R2 | R1 | R2 | R1 | R2 | R1 | R2 | R1 | R2 |
| **Padrauna** | Bahadurganj | 0 | 0 | 0 | 0 | 0 | 0 | 0 | 0 | 0 | 0 | 0 | 22 | 1 | 4 |
|  | Sarrhie | 0 | 0 | 0 | 0 | 0 | 0 | 0 | 0 | 0 | 0 | 1 | 0 | 5 | 3 |
|  | Pipra Majra | 0 | 0 | 0 | 0 | 0 | 0 | 0 | 0 | 0 | 0 | 1 | 12 | 3 | 3 |
|  | Sidhua | 0 | 0 | 0 | 0 | 0 | 0 | 0 | 0 | 0 | 0 | 0 | 1 | 5 | 2 |
| **Kaptanganj** | Amdiha | 1 | 0 | 0 | 0 | 0 | 0 | 0 | 0 | 0 | 0 | 0 | 4 | 2 | 3 |
|  | Gajara | 0 | 2 | 0 | 0 | 0 | 0 | 0 | 0 | 0 | 0 | 24 | 6 | 2 | 3 |
|  | Ghurahupur | 0 | 0 | 0 | 0 | 0 | 0 | 0 | 0 | 0 | 0 | 70 | 8 | 1 | 3 |
|  | Magdiha | 1 | 0 | 0 | 0 | 0 | 0 | 0 | 0 | 0 | 1 | 0 | 1 | 4 | 4 |
| **Khadda** | Belwa Jungal | 1 | 1 | 0 | 0 | 0 | 0 | 0 | 0 | 0 | 0 | 70 | 1 | 2 | 3 |
|  | Bulahwa | 1 | 1 | 0 | 0 | 0 | 0 | 0 | 0 | 0 | 3 | 22 | 5 | 4 | 5 |
|  | Chamar Diha | 2 | 3 | 0 | 0 | 0 | 0 | 0 | 0 | 0 | 0 | 0 | 1 | 1 | 4 |
|  | Patkhauli | 0 | 1 | 0 | 0 | 0 | 0 | 0 | 0 | 0 | 0 | 7 | 16 | 3 | 4 |
|  |  | 6 | 8 | 0 | 0 | 0 | 0 | 0 | 0 | 0 | 4 | 195 | 77 | 33 | 41 |
