# Supplementary material for: Identifying sources, pathways and risk drivers in ecosystems of Japanese Encephalitis in an epidemic-prone north Indian district
Source: PLoS One. 2017 May 2;12(5):e0175745. doi: 10.1371/journal.pone.0175745 (PMC5412994; doi:10.1371/journal.pone.0175745)
Supplement: S3 Table — (DOCX) [file pone.0175745.s003.docx]

## Table S3: Number of mosquitoes collected from the peridomestic biotope

|  |  | ***Culex vishnui*** | | ***Culex tritaeniorhynchus*** | | ***Culex gelidus*** | | ***Culex epidesmus*** | | ***Culex whitmorei*** | | **Other mosquito species** | | **Number of sites sampled** | |
| --- | --- | --- | --- | --- | --- | --- | --- | --- | --- | --- | --- | --- | --- | --- | --- |
|  |  | R1 | R2 | R1 | R2 | R1 | R2 | R1 | R2 | R1 | R2 | R1 | R2 | R1 | R2 |
| **Padrauna** | Bahadurganj | 0 | 2 | 0 | 0 | 0 | 0 | 0 | 0 | 0 | 0 | 0 | 15 | 3 | 9 |
|  | Sarrhie | 0 | 0 | 0 | 0 | 0 | 0 | 0 | 0 | 0 | 0 | 1 | 7 | 10 | 12 |
|  | Pipra Majra | 0 | 0 | 0 | 0 | 0 | 0 | 0 | 0 | 0 | 0 | 3 | 2 | 7 | 9 |
|  | Sidhua | 2 | 0 | 0 | 0 | 0 | 0 | 0 | 0 | 0 | 0 | 2 | 7 | 10 | 10 |
| **Kaptanganj** | Amdiha | 1 | 0 | 0 | 0 | 0 | 0 | 0 | 0 | 0 | 0 | 5 | 6 | 3 | 9 |
|  | Gajara | 2 | 0 | 0 | 0 | 0 | 0 | 0 | 0 | 0 | 0 | 0 | 5 | 4 | 8 |
|  | Ghurahupur | 1 | 0 | 0 | 0 | 0 | 0 | 0 | 0 | 0 | 0 | 397 | 7 | 3 | 7 |
|  | Magdiha | 1 | 0 | 0 | 0 | 0 | 0 | 0 | 0 | 0 | 0 | 2 | 1 | 4 | 5 |
| **Khadda** | Belwa Jungal | 1 | 1 | 0 | 0 | 0 | 0 | 0 | 0 | 0 | 0 | 0 | 8 | 6 | 13 |
|  | Bulahwa | 0 | 2 | 0 | 0 | 0 | 0 | 0 | 0 | 0 | 1 | 0 | 6 | 3 | 9 |
|  | Chamar Diha | 0 | 3 | 0 | 0 | 1 | 0 | 0 | 1 | 1 | 1 | 30 | 22 | 5 | 11 |
|  | Patkhauli | 1 | 0 | 0 | 0 | 0 | 0 | 0 | 0 | 0 | 0 | 0 | 7 | 7 | 7 |
|  |  | 9 | 8 | 0 | 0 | 1 | 0 | 0 | 1 | 1 | 2 | 440 | 93 | 65 | 109 |
