# Supplementary material for: Identifying sources, pathways and risk drivers in ecosystems of Japanese Encephalitis in an epidemic-prone north Indian district
Source: PLoS One. 2017 May 2;12(5):e0175745. doi: 10.1371/journal.pone.0175745 (PMC5412994; doi:10.1371/journal.pone.0175745)
Supplement: S4 Table — (DOCX) [file pone.0175745.s004.docx]

# Table S4: List of quantitative and qualitative data collection methods, sampling scheme and study population

|  | METHODS | SAMPLING SCHEME | POPULATION |
| --- | --- | --- | --- |
| QUANTITATIVE ARM | Household (HH) surveys | 5% HH from village | Heads of household |
|  | Human blood sample collection | All subjects 1- 15 years in sampled HH | Children |
|  | Pig blood sample collection | Up to 16 pigs each from 6 pig-owning villages, 2-6 months old | Pigs |
|  | Vector sample collection and survey | Collection from village clusters in domestic indoor, domestic outdoor and peri domestic biotopes | Mosquitoes |
|  | Remote sensing on land use/land cover (LU/LC) patterns | Secondary satellite data of land-use and land-cover (vegetation and road layers) | District-level |
|  | GPS surveys | GPS locations of breeding grounds, relevant ecological landmarks, household and animal holding locations, educational facilities, healthcare facilities, administrative units, and breeding sites from 3 blocks | Village-level |
| QUALITATIVE ARM | In-depth interviews | Non-probability purposive sample from each of 3 blocks till saturation | Pig owners  Utilisers of acute care for AES  NGO/CBO  Healthcare provider (Human) (ANM, MO)  Health care provider (Veterinary) (LEO, BVO)  District level provider (Human health & veterinary) |
|  | Focus group discussions | Non-probability purposive sample from each of 3 blocks till saturation | Farmers, Community members,  Students |
