# Supplementary material for: Identifying sources, pathways and risk drivers in ecosystems of Japanese Encephalitis in an epidemic-prone north Indian district
Source: PLoS One. 2017 May 2;12(5):e0175745. doi: 10.1371/journal.pone.0175745 (PMC5412994; doi:10.1371/journal.pone.0175745)
Supplement: S5 Table — (DOCX) [file pone.0175745.s005.docx]

# Table S5: Main Variables Studied in Quantitative Analysis

| Human | 1. Demographics:    1. Gender    2. Religion and caste    3. Literacy    4. Primary occupation    5. Land ownership    6. Crops grown on farmed land    7. Time spent in agricultural activities in the fields; time spent by children <15 years in family in fields    8. Past history of or current employment in brick kilns    9. Time spent in brick kilns; time spent by children <15 years in family in brick kilns    10. Socioeconomic status based on ownership of commodities 2. Housing    1. Ownership status, living rooms, room use details    2. Structure of house: pucca, semi pucca or kuchha    3. Flooring material    4. Roofing material    5. Exterior walls    6. Windows    7. Lighting    8. Fuel used generally    9. Water for drinking, cooking and washing: Source, storage, purification practices    10. Indoor toilet facilities and usage practices    11. Handwashing practices 3. Children    1. Behavioral pattern to assess risk of exposure to vectors in different seasons, at different times of the day    2. Vaccination status for JE    3. Previous history of suffering from a known episode of JE    4. Family history of someone suffering from a known episode of JE |
| --- | --- |
| Animal | 1. Animal ownership pattern, including species and numbers owned 2. Purpose of keeping animals 3. Housing status of animals 4. Grazing practices of animals 5. Distance between animal and human habitations 6. Pig Specific:    1. Number, purpose    2. Shelter details    3. For individual pig: age, gender, farrow history, piglet status, vaccination status, reasons for non-vaccination, shelter type lived in, distance of shelter from human sleeping/living rooms, roaming habits of pig, time spent in human contact in family, time spent in fields. |
| Vectors | 1. Behavioral pattern to assess risk of exposure to vectors in different seasons, at different times of the day 2. Known breeding source in vicinity of residence 3. Indoor residual spraying of pesticides in the past year 4. Slef reported mosquito net ownership and usage patterns, including treatment with pesticides 5. Vector Collection    1. Site of collection    2. Biotope details: Domestic       1. GPS location       2. Number of rooms where humans and/or animals    3. Biotope Details: Peridomestic       1. Distance from village center/domestic biotopes       2. GPS Location, height of sampling site       3. Nature of sampling site    4. Water sample:       1. Distance from village center/domestic biotopes       2. GPS Location of sampling site       3. Distance from nearest home       4. Type, shape, size, depth and soil in water body       5. Algal and emergent plant coverage       6. pH, turbidity, dissolved oxygen |
