# Supplementary material for: Identifying sources, pathways and risk drivers in ecosystems of Japanese Encephalitis in an epidemic-prone north Indian district
Source: PLoS One. 2017 May 2;12(5):e0175745. doi: 10.1371/journal.pone.0175745 (PMC5412994; doi:10.1371/journal.pone.0175745)
Supplement: S6 Table — (DOCX) [file pone.0175745.s006.docx]

# Table S6: List of Drivers by Biotopes

| **Domestic Indoor** | **Domestic Outdoor** | **Peri-Domestic** | **Systems** |
| --- | --- | --- | --- |
| Predominant vector species in domestic indoor is *Culex vishnui* | High preference of vectors for human blood compared to animal blood. | *C*. *vishnui* prefers resting in mosaic crop vegetation | Vaccination rates seen to correspond with infection rates. |
| High indoor density and preference for human blood in *C. vishnui* | Skewed human:animal ratio increases zoopotentiation without providing zooprophylaxis. | Area under paddy cultivation | Awareness about disease, causes and treatment options low, increasing risks |
| *C. whitmorei* exhibiting similar resting and feeding pattern as *C. vishnui* | Higher vector density in immediate domestic outdoor compared to peri-domestic biotope | Mixed cropping system seen to have protective effect | Utilization of healthcare services poor and the first point of contact is usually traditional healers |
| Emerging endophagic and endophilic biting behavior in vectors | High prevalence of JE positivity in pigs which act as amplifying hosts |  |  |
| Indoor residual spraying observed to have protective effect. | Children sleeping outdoors |  |  |
