# Supplementary material for: Identifying sources, pathways and risk drivers in ecosystems of Japanese Encephalitis in an epidemic-prone north Indian district
Source: PLoS One. 2017 May 2;12(5):e0175745. doi: 10.1371/journal.pone.0175745 (PMC5412994; doi:10.1371/journal.pone.0175745)
Supplement: S7 Table — (DOCX) [file pone.0175745.s007.docx]

# Table S7: List of Villages by Pig Ownership

| Blocks | Pig Owning Villages | Non-Pig Owning Villages |
| --- | --- | --- |
| Padrauna | Sandi Bujurg  Sidhua | Bahadurganj  Dharampur Bujurg |
| Kaptanganj | Amdiha  Gajara | Ghurahupur  Magdiha |
| Khadda | Bulahwa  Chamardiha | Basdila  Juda Chhapra |
