# Supplementary material for: Identifying sources, pathways and risk drivers in ecosystems of Japanese Encephalitis in an epidemic-prone north Indian district
Source: PLoS One. 2017 May 2;12(5):e0175745. doi: 10.1371/journal.pone.0175745 (PMC5412994; doi:10.1371/journal.pone.0175745)
Supplement: S8 Table — (DOCX) [file pone.0175745.s008.docx]

# Table S8: Man:Animal Ratios in Study Villages

| Block | Village | Man:Bovine ratio | Man:Animal ratio | Man:Goat |
| --- | --- | --- | --- | --- |
| Padrauna | Bahadurganj | 21 | 7 | 10.50 |
|  | Sarrhie | 14.2 | 8.9 | 23.67 |
|  | Pipra Majra | 7.3 | 4.8 | 13.75 |
|  | Sidhua | 18 | 5.4 | 7.71 |
| Kaptanganj | Amdiha | 11.6 | 9.1 |  |
|  | Gajara | 33 | 33 |  |
|  | Ghurahupur | 29 | 29 |  |
|  | Magdiha | 6.6 | 6.6 |  |
| Khadda | Belwa Jangal | 36 | 18 | 36.00 |
|  | Bulahwa | 70 | 5.8 | 7.00 |
|  | Chamar Diha | 8.3 | 3.4 | 5.77 |
|  | Patkhauli | 8.8 | 6.8 | 29.33 |
