# Supplementary material for: Identifying sources, pathways and risk drivers in ecosystems of Japanese Encephalitis in an epidemic-prone north Indian district
Source: PLoS One. 2017 May 2;12(5):e0175745. doi: 10.1371/journal.pone.0175745 (PMC5412994; doi:10.1371/journal.pone.0175745)
Supplement: S1 File — (DOCX) [file pone.0175745.s009.docx]

# File S1: Details of Ethical Approvals

The study received ethical approval from PHFI’s Institutional Ethics Committee. The Institute Animal Ethics Committee of the Indian Veterinary Research Institute (IVRI) approved the animal component of the study (Approval #10, dated 2 Dec 2011, IVRI IAEC proceedings) as per the guidelines developed by the Committee for the Purpose of Control and Supervision of Experiments on Animals. Written informed consent was obtained from adult respondents in each household selected for household surveys. Consent was obtained from children orally since a written consent was not obtainable because they could not sign their names. However, written assent was taken from the parents of the child after informing them about the research study, including risks and possible benefits of participating in the study. Similarly, written informed consent was obtained from pig owners. All participants were told they could withdraw consent at any point before/during the interview process. Entomological sampling was done on private land and inside private residences after obtaining written informed consent from the owners.

Quality assurance measures were instituted by the ERCG through standardization of protocols, intense training and monitoring of data collectors to assure data quality, and systematic data management through standardized coding and labelling of specimens, while linking all elements in a relational transdisciplinary database.
